# 733. Carbapenem-Resistant Enterobacterales (CRE) Colonization Prevalence in Botswana: an Antibiotic Resistance in Communities and Hospitals (ARCH) Study

**DOI:** 10.1093/ofid/ofab466.930

**Published:** 2021-12-04

**Authors:** Naledi Mannathoko, Mosepele Mosepele, Rachel Smith, Robert Gross, Laurel Glaser, Kevin Alby, Melissa Richard-Greenblatt, Aditya Sharma, Anne Jaskowiak, Kgotlaetsile Sewawa, Emily Reesey, Laura Cowden, Leigh Cressman, Dimpho Otukile, Giacomo Paganotti, Margaret Mokomane, Ebbing Lautenbach

**Affiliations:** 1 University of Botswana, Gaborone, South-East, Botswana; 2 Centers for Disease Control and Prevention, Decatur, GA; 3 University of Pennsylvania, Phiadelphia, Pennsylvania; 4 University of North Carolina at Chapel Hill School of Medicine, Chapel Hill, North Carolina; 5 Botswana UPenn Partnership, Gaborone, South-East, Botswana; 6 University of Pennsylvania School of Medicine, Philadelphia, Pennsylvania

## Abstract

**Background:**

Although CRE are a global threat, data in low- and middle-income countries are scarce. Colonization data are vital for informing antibiotic resistance strategies. We characterized the colonization prevalence of CRE in various settings in Botswana.

**Methods:**

This study was conducted in 3 districts in Botswana (1 hospital and 2 clinics per district). Adult inpatients and clinic patients were randomly selected for enrollment. Community subjects were enrolled by inviting each enrolled clinic subject to refer up to 3 adults. Each adult clinic or community subject was also asked to refer their children. All subjects had rectal swabs obtained and inoculated on selective chromogenic media for preliminary identification of CRE. Final identification and susceptibility testing were performed using MALDI-TOF MS and VITEK-2, respectively. CRE underwent genotyping for carbapenemase genes.

**Results:**

Subjects were enrolled from 1/15/20-9/4/20 with a pause from 4/2/20-5/21/20 due to a countrywide COVID lockdown. Of 5,088 subjects approached, 2,469 (49%) participated. Enrollment by subject type was: hospital – 469 (19%); clinic – 959 (39%); community adult – 477 (19%); and community child – 564 (23%). Of 2,469 subjects, the median (interquartile range) age was 32 years (19-44) and 1,783 (72%) were female. 42 (1.7%) subjects were colonized with at least one CRE; 10 subjects were colonized with multiple strains. *E. coli* (n=17), *K. pneumoniae* (n=20), and *E. cloacae* complex (n=11) were most common. CRE colonization prevalence was 6.8% for hospital subjects, 0.7% for clinic subjects, 0.2% for adult community subjects, and 0.5% for child community subjects (p< 0.001)). CRE prevalence varied across regions (Figure 1) and was significantly higher pre- vs post-lockdown (Figure 2). VIM and NDM were the most common carbapenemase genes (Figure 3).

Figure 1. CRE Colonization - Study Sites

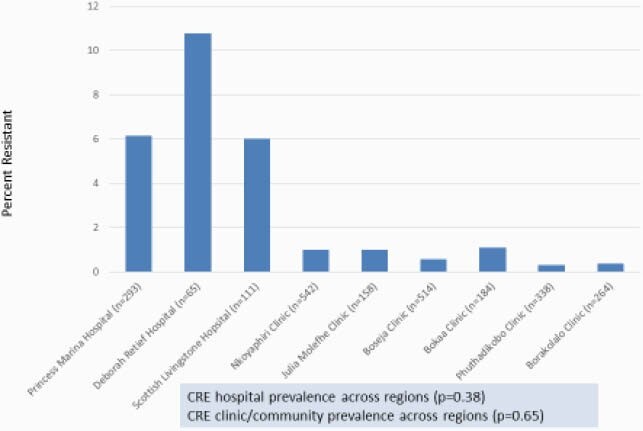

Figure 2. CRE Colonization - Temporal Trends

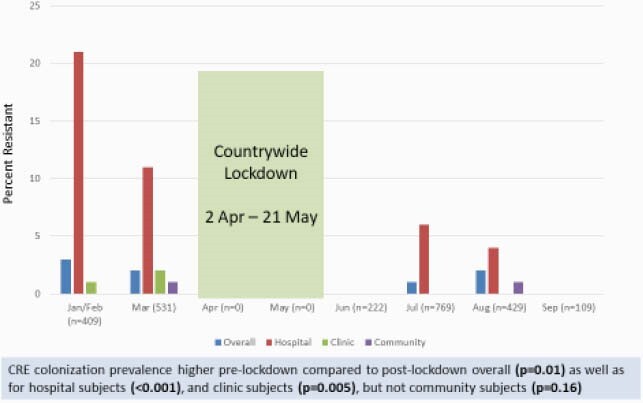

FIgure 3. CRE Genotypic Analyses

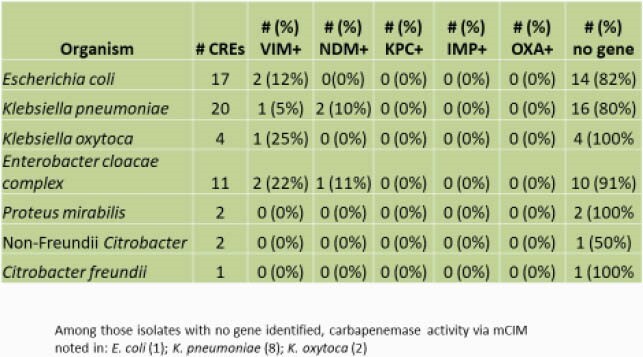

**Conclusion:**

CRE colonization was significantly higher in hospital vs community settings in Botswana. CRE prevalence varied by region and decreased significantly following a countrywide lockdown. With CRE prevalence still modest, elucidating risk factors for CRE colonization holds promise in developing strategies to curb further emergence of CRE. Additional investigation of the CRE isolates without identified resistance genes is warranted.

**Disclosures:**

**Robert Gross, MD, MSCE**, **Pfizer** (Other Financial or Material Support, Serve on DSMB for drug unrelated to HIV) **Ebbing Lautenbach, MD, MPH, MSCE**, **Merck** (Other Financial or Material Support, Member of Data and Safety Monitoring Board (DSMB))

